# Comparison between Short Therapy and Standard Therapy in Pediatric Patients Hospitalized with Urinary Tract Infection: A Single Center Retrospective Analysis

**DOI:** 10.3390/children9111647

**Published:** 2022-10-28

**Authors:** Danilo Buonsenso, Giorgio Sodero, Francesco Mariani, Ilaria Lazzareschi, Francesco Proli, Giuseppe Zampino, Luca Pierantoni, Piero Valentini, Claudia Rendeli

**Affiliations:** 1 Department of Woman and Child Health and Public Health, Fondazione Policlinico Universitario Agostino Gemelli IRCCS, 00168 Rome, Italy; 2 Centro di Salute Globale, Università Cattolica del Sacro Cuore, 00168 Roma, Italy; 3 Pediatric Emergency Unit, IRCCS Azienda Ospedaliero-Universitaria di Bologna, 40138 Bologna, Italy

**Keywords:** urinary tract infection, children, short antibiotic therapy

## Abstract

Introduction: There is marked heterogeneity in clinicians’ choice of antibiotic duration for pediatric urinary tract infections (UTIs). Most patients with bacterial UTIs still receive between 7 and 10 days of antibiotics. Prolonged antibiotic exposure drives the emergence of resistance and increases the occurrence of adverse effects. There is increasing evidence that shorter antibiotic regimens may be equally effective compared with longer ones. However, studies evaluating shorter therapies in children hospitalized with urinary tract infections have not yet been performed. Methods: We performed a retrospective study comparing children hospitalized with UTIs treated with a short antibiotic (<7 days) or standard antibiotic treatment. The primary aim of our study was to assess the efficacy of a shorter antibiotic therapy for children with UTIs, compared with an historical group of children treated with a standard 7–14 days course. Results: 112 patients, 46 of which were females (41.1%) with a median age 6 months were enrolled. A total of 33 patients (29.5%) underwent a short therapy. All patients were successfully discharged from the acute episode, independently from antibiotic duration. Short therapy was associated with a lower risk of urinary tract relapse (22 relapses (95.6%) in the standard group, 1 (4.4%) in the short group; OR 0.081; 95%CI 0.01–0.63). Conclusions: Short antibiotic therapy was equivalent to standard duration therapy for the cure of UTIs in hospitalized children and was also associated with a lower rate of recurrences. This study provides the basis for a larger prospective randomized study to address the role of short antibiotic therapies in children with UTIs requiring hospitalization

## 1. Introduction

Urinary tract infection (UTI) is a common problem in the pediatric population [1], with up to 2% of males and 8% of females experiencing at least one episode of UTI by the age of approximately six years [2]. Under two years of age, the prevalence of acute pyelonephritis in a child with fever of unknown origin is around 5%, without distinction between females and uncircumcised males. Gram-negative bacteria are responsible for more than 95% of pyelonephritis, mainly *Escherichia coli,* followed by *Klebsiella pneumoniae, Proteus mirabilis, Enterobacter* and *Citrobacter*, while infections due to Gram-positive bacteria are rare [2,3].

To date, there is marked heterogeneity in the clinicians’ choice of antibiotic duration for pediatric UTIs. Most patients with bacterial UTIs still receive between 7 and 10 days of antibiotics, although these durations are based largely on expert opinion, and recent evidence from adult studies and a randomized trial indicate clinical noninferiority between 7-day and 14-day courses [4,5]. Fixed antibiotic durations provide straightforward guidance but do not consider host characteristics or treatment response, and they are not in line with the modern concept of personalized medicine. Importantly, prolonged antibiotic exposure drives the emergence of resistance and increases the occurrence of adverse effects, such as *Clostridioides difficile infection* and other microbiota disruptions, and longer courses of antibiotics are also associated with lengthier hospitalizations and higher costs [6,7]. In addition, antibiotic exposure during early life periods can also alter the gut microbiota which, according to recent studies, is critical to shape proper immunological responses in children and balance between defense and self-recognition [8].

For these reasons, there is a growing interest in shorter antibiotic regimens to treat common infections. A recent pediatric retrospective observational study aimed to evaluate the effectiveness of a short-course antibiotic regimen in children with UTIs [9]. In this study involving 791 children aged 6 months to 18 years (37.5% were prescribed a short course and 62.5% a prolonged course of antibiotics), the odds of treatment failure were similar for patients prescribed a short course vs. a prolonged course of antibiotics (11.2% vs. 9.4%; odds ratio, 1.22; 95% CI, 0.75–1.98). There was no significant difference in the odds of a drug-resistant uropathogen for patients with a subsequent urinary tract infection within 30 days when prescribed a short course vs. a prolonged course of antibiotics (40% vs. 64%; odds ratio, 0.36; 95% CI, 0.09–1.43). However, this study did not included infants younger than 6 months of age, which are a population at higher risk of UTIs, and considered a relatively long period of antibiotic duration (6–9 days) as a short day course, which is longer than the recent evidence of adult studies showing that 5 days can be enough in several bacterial infections, from hospital to community-acquired pneumonia [10]. 

In our center, since recent improved understanding about the direct and indirect benefits of a shortened antibiotic course [11], we implemented a number of local antimicrobial stewardship programs, which also include a protocol for a short treatment regimen of <7 days for children admitted with uncomplicated febrile UTI (negative blood cultures and a lack of urinary tract malformation). This allowed us to perform this retrospective study aiming to assess the efficacy of a shorter antibiotic therapy for children with UTIs, compared with an historical group of children treated with a standard 7–14 days course.

## 2. Methods 

This is a retrospective study of children aged more than 60 days up to 17 years who were admitted in the pediatric wards for at least 3 days with a diagnosis of UTI from March 2017 to April 2022. All patients were followed up, as per routine clinical practice in our center, for a period of ninety days after admission to evaluate for UTI relapses based on clinical and microbiological definitions, in our outpatient service of Pediatric Urologic Disorders. Follow-up data were collected by the outpatient charts of our outpatient service of Pediatric Urologic Disorders. The time for shifting from intravenous to oral antibiotic was subjective according to the attending physician. In our center, local guidance suggests intravenous amoxicillin/clavulanate empiric therapy for children admitted with a UTI, or ceftriaxone for ill-appearing children; once antibiogram is available, antibiotic treatment is decided according to susceptibility and safety/toxicity. Antibiotic duration was calculated by the day that the effective susceptible antibiotic was used (for example, if a child is treated with empirical amoxicillin/clavulanate and the urine culture shows a susceptible bacteria, than day one is considered from the first day of amoxicillin/clavulanate use; if the child is treated with empirical amoxicillin/clavulanate and the urine culture shows a resistant bacteria and the antibiotic is changed, day one is considered from the beginning of the new effective antibiotic). The duration of antibiotic therapy was decided by the evaluating physician in our pediatric ward, which included four pediatricians, two of them being specialized in pediatric infectious diseases and part of the antibiotic stewardship committee of our hospital providing guidance of shortening antibiotic therapies as new evidence is found. We define a short antibiotic duration when it lasts < 7 days, and standard if lasting 7 days or more.

In our center, reasons for admission for a UTI are: being unable to retain fluids; dehydration requiring intravenous fluids; ill-appearing/sepsis-like status.

### 2.1. Inclusion Criteria

- Age > 60 days.
- Clinical diagnosis of febrile UTI defined by a fever of ≥38 °C AND:
- A positive result of urinalysis (nitrite and/or leukocyte esterase positivity) in one urine sample collected by bladder catheterization or clean-voided urine AND positive urine culture for a single type of bacterium with a charge of >104 CFU/mL (or, in the case of more than one germ isolated with at least one of them having a charge of >104 CFU/mL).
- Positive urine culture for a single type of bacterium with a charge of >104 CFU/mL, even in the case of normal urinalysis if the child had compatible signs and symptoms and blood laboratory findings AND no other alternative diagnoses were obtained AND the treating clinicians considered the case and treated as a UTI AND the child was discharged with only the diagnosis of a UTI.
- A clinical diagnosis of afebrile UTI defined by compatible signs and symptoms (e.g., difficulty → feeding, vomiting, irritability) AND:

A positive result of urinalysis (nitrite and/or leukocyte esterase positivity) in one urine sample collected by bladder catheterization or clean-voided urine AND positive urine culture for a single type of bacterium with a charge of >104 CFU/mL (or, in the case of more than one germ isolated with at least one of them having a charge of >104 CFU/mL).

- Admitted because of UTI.
- No urinary tract abnormalities.
- Negative blood cultures.

### 2.2. Exclusion Criteria

- Antibiotic administration in the 3 days prior to admission.
- Age > 18 years.
- Immune deficiencies.
- At least one of the following urinary tract abnormalities:
  - Unilateral or bilateral renal hypoplasia.
  - Pelvi-caliceal dilatation (2° SFU grade hydronephrosis).
  - Ureteral dilatation.
  - Bladder abnormalities (ureterocele, diverticulum).

## 3. Outcomes

The primary outcome was to address the UTI relapse rate in children treated with a short (<7 days) or long course of antibiotic therapy (≥7 days).

Secondary outcomes were:

- To evaluate the impact of clinical, demographic and microbiological characteristics on UTI relapse in the two groups of children treated with a short or long course of antibiotic therapy.
- To evaluate the impact of a short or long course of antibiotic therapy on a relapse with a similar bacteria or a bug resistant to the originally used antibiotic.
- To evaluate relapse rates over time during a 90-day period of follow-up.
- A comparison between the characteristics of relapse patients and non-relapse patients.

## 4. Statistical Analysis

For continuous variables, the Kolmogorov–Smirnov test was used to assess normal distribution. Categorical variables were reported as count and percentage. Continuous variables with normal distribution were expressed as mean with standard deviation; data with skewed distribution were expressed as median with interquartile range (IQR). Categorical variables were compared by Chi-squared tests or Fisher’s exact tests and the strength of this association, when significant, was evaluated considering the “V” of Cramer. A Student’s *t*-test or Mann–Whitney U test was used to assess differences in the two groups for continuous variables as appropriate. A *p* value of <0.05 was considered statistically significant.

To evaluate the impact of the short therapy on the risk of relapse, odds ratio and 95% confidence intervals (OR, 95% CI) were calculated. 

Kaplan–Meyer curves were built to compare patients who underwent standard therapy and patients who underwent short therapy. A log rank test was performed to evaluate if there was a statistically significant difference between the two curves. 

The statistical analysis was performed using IBM SPSS Statistics 26.0 software (IBM Corporation, Armonk, NY, USA).

### Ethic Committee

The study was reviewed and approved by the Human Research Ethics Committee of the Fondazione Policlinico Universitario A. Gemelli IRCCS of Rome, Italy (prot 0010122/22, ID 4808). The study was conducted in accordance with the Declaration of Helsinki and its subsequent amendments. No personal or identifiable data were collected during the conduct of this study. 

## 5. Results

### 5.1. Study Population

During the study period, 181 children were admitted for a clinical diagnosis of a UTI; of them, 37 were excluded due to urological abnormalities, 2 due to age, 21 due to a negative urine culture and 9 due to concomitant bacteremia. Therefore, 112 patients, 46 of which were females (41.1%), were enrolled in our study. Fifteen of these (13.4%) were affected by other comorbidities (one orchiepididymitis, three SARS-CoV-2 infection, two asthma, six bronchiolitis, one atrial defect) and 13 patients (11.7%) had a renal echography pathological (a single patient did not perform the urinary tract echography). Four children (3.6%) had a prenatal echography that reported an alteration. Upon admission, 110 children (98.2%) had fever, 83 (74.1%) had alimentary difficulties, 40 (35.7%) had vomiting, 21 (18.8%) had diarrhea and 33 (29.5%) had other symptoms. The characteristics of the population are reported in Table 1.

Regarding the method of urine collection, in 102 cases (91.1%) urine was collected by catheterization, in six cases (5.4%) by clean-void catch and in four cases (3.6%) by urinary bag. Urinary test results showed leukocyte esterase in 91 patients (81.3%), nitrites in 49 patients (43.8%) and both leukocyte esterase and nitrites in 45 patients (40.2%). Regarding the urine culture results, *Escherichia coli* was the most frequently isolated microorganism (88 times, 78.6%), followed by *Enterococcus faecalis*, *Klebsiella pneumoniae*, *Pseudomonas aeruginosa* and *Proteus mirabilis*. Concerning antibiotic resistance, 19 (17.0%) germs were resistant to amoxicillin-clavulanate, 15 (13.4%) to cephalosporins, 13 (11.6%) to the antibiotic used for the empirical treatment and 39 (34.8%) to another antibiotic.

Blood exams were performed for most of the patients at admission. White blood cell count resulted abnormal in 99 cases (88.4%). C-reactive protein was dosed in 110 patients (98.2%) and resulted pathological (>5 mg/L) in 100 patients (89.3%), while procalcitonin was dosed in 44 cases (39.3%) and tested positive (>0.05 ng/mL) in 10 cases (8.9%). The median value of CRP was 40.15 (93.4) mg/L, while the median value of PCT was 0.16 (0.78) ng/mL. 

For the empirical treatment, 83 patients (74.1%) were treated with a single antibiotic, while the remaining 29 (25.9%) underwent a two-fold antibiotic therapy. The antibiotic most frequently administered was amoxicillin-clavulanate in 47 patients (42%); 29 infections (25.9%) were treated with ceftriaxone, 21 (18.8%) with the association of ampicillin and amikacin and 15 (13.4%) with another antibiotic.

In our cohort, 35 patients (31.3%) modified the antibiotic therapy in progress due to medical decision (e.g., selecting an antibiotic with an oral option available). For what concerns the duration of antibiotic therapy, 33 patients (29.5%) underwent a short therapy (less than 7 days of effective antibiotic, of which only four (12.1%) were treated for 6 days and the remaining patients for 5 days). The median duration of short therapy was 5 (0) days, while the median duration of standard therapy was 7 (3) days.

All patients were successfully discharged from the acute episode, independently from antibiotic duration.

### 5.2. Duration of Antibiotic Therapy and Outcomes

A total of 23 relapses (20.5%) were reported during the study period and nine of them required hospitalization (8%). A total of 5 (4.5%) patients presented a relapse within 7 days from discharge, 10 patients (8.9%) within 30 days, 5 patients (4.5%) within 60 days and 3 patients (2.7%) within 90 days. 

**Figure 1:**
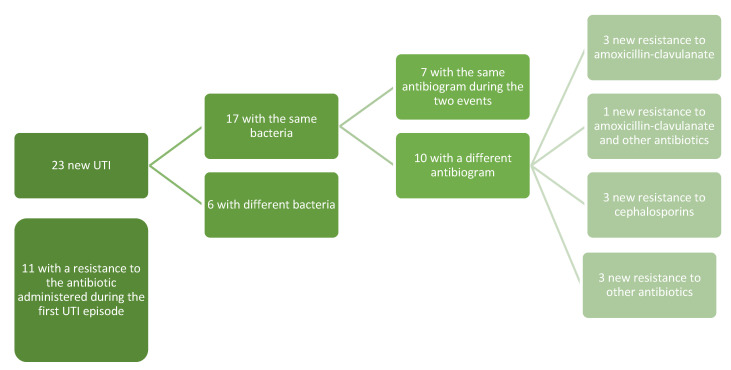
Pattern of the resistances of the first and relapsed UTIs.

Seventeen of the relapses (17/23, 73.9%) were by the same microorganism of the first infection, while six relapses were from a different bacterium. Overall, independently from the bacteria involved in the first UTI and in the relapse, in 11 relapses (47.8%) the infection was due to a microorganism with resistance to the antibiotic administered during the first infection (Figure 1).

**Figure 2:**
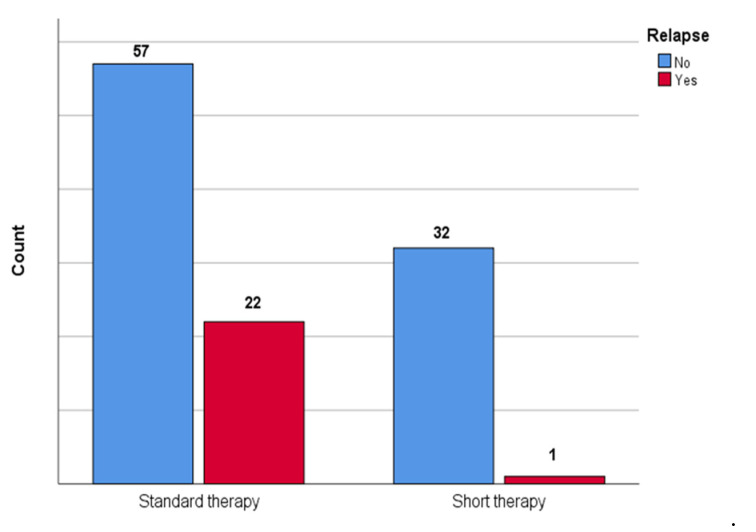
The figure shows the comparison between patients who underwent short therapy and patients who underwent standard therapy in the number of urinary tract relapses in our cohort.

Figure 2 shows the comparison between patients who underwent short therapy and patients who underwent standard therapy in terms of relapse; a statistically significant difference between groups was observed (*p* = 0.002) with lower association (V = 0.28) in the short therapy group.

A comparison between the characteristics of patients with relapse and patients without relapse is reported in Table 2. The only statistically significant association observed was with the neutrophil count.

Table 3 shows the comparison of the first urinary tract infection characteristics between patients with relapse and patients without relapse. No statistically significant association was observed. 

The Mann–Whitney test showed a statistically significant difference in the number of antibiotic therapy days, which was higher in patients who had a relapse (*p* = 0.001). 

To evaluate the impact of the short therapy on the risk of relapse, the odds ratio was calculated; in our sample it was observed how short therapy was associated with a lower risk of urinary tract relapse (OR 0.081; 95% CI 0.01–0.63).

**Figure 3:**
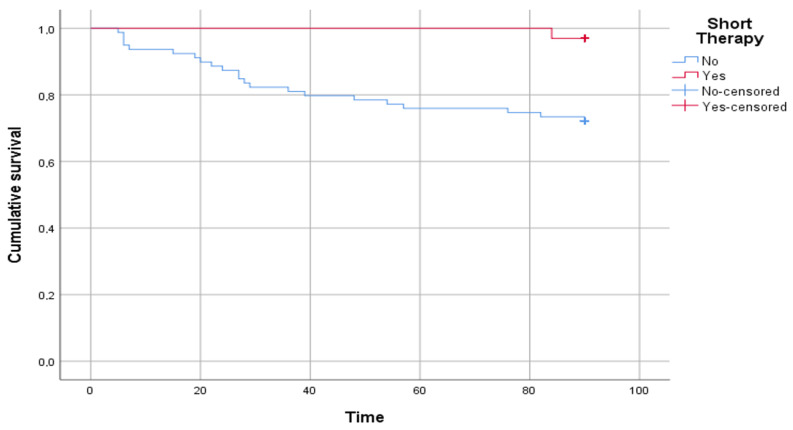
The figure shows the comparison between patients who underwent short therapy and patients who underwent standard therapy in time free from disease, intended as a relapse of urinary tract infection. The time on the abscissa axis is expressed in days.

Figure 3 shows the results of the survival analysis. A statistically significant difference was reported in the time free from disease between patients who underwent short therapy and patients who underwent standard therapy (log rank *p* = 0.004). 

## 6. Discussion

In this study, we compared two historical cohorts of children with a UTI severe enough to require hospitalization and treated with a short (<7 days) vs. standard length (≥7 days) of antibiotic therapy, showing a lower rate of UTI relapses in children treated with a short course. To our knowledge, this is the first study addressing the role of shorter antibiotic therapy on time-free UTI relapses in young, hospitalized children with UTIs.

The duration of antibiotic therapy in pediatric patients due to UTIs is not standardized and, in most cases, is prolonged for up to at least 7 days of treatment, reaching 14 days in severe cases or in patients at a higher risk of complications [3]. Hospitalized children are typically unable to receive oral drugs or on suspicion of urosepsis, and receive intravenous antibiotic therapy [12], although it is equivalent, in terms of effectiveness, to oral therapy [13]; similarly, hospitalized patients usually receive longer antibiotic therapies than non-hospitalized children [14].

Different guidelines on the treatment of pediatric UTIs are not uniform regarding the duration of antibiotic therapy; according to the EAU/ESPU guidelines, [12] a treatment period of 7–14 days is better than a short treatment period (1–3 days of therapy) in preventing complications such as renal scarring and the systemic dissemination of infection, although an intermediate treatment period (up to 5 days) can be effective in low urinary tract infections. However, in young infants it can be challenging to differentiate between high vs. low UTIs. NICE guidelines [15] recommend at least 10 days of antibiotic therapy, administered orally, in children older than 3 months with suspicion of pyelonephritis, while short therapy (3 days) is recommended only if there is a high probability of lower urinary tract infection. The guidelines of the Italian Society of Pediatric Nephrology [16] establish a minimum therapy of 10 days, which is administered orally in uncomplicated cases or intravenously in pyelonephritis, also prolonged up to 14 days in urosepsis; short therapy is considered only as a “parenteral short”, with the transition from intravenous to oral therapy. The same guidelines advise against the use of antibiotic prophylaxis following the first episode of urinary tract infection. The American Academy of Pediatrics [17] establishes that there are no definitive data about the duration of therapy in children with febrile urinary tract infections or pyelonephritis, although it recognizes that children exposed to antibiotic doses for longer periods (for example, during antibiotic prophylaxis), may develop a higher rate of future resistant germ infections.

In clinical practice, the average duration of treatment for urinary tract infections is at least 7 days, regardless of the type of antibiotic chosen or the route of administration [3]. In our cohort of pediatric patients, children with UTIs who underwent antibiotic therapy for a shorter duration (≤5 days) had a significantly lower probability of experiencing a UTI recurrence (1 versus 22, OR 0.081; 95% CI 0.01–0.63) than patients treated for the standard duration. Interestingly, we also found statistically significant differences in the relapsed UTI-free time between short and standard antibiotic courses (*p* = 0.004). Although the different guidelines marginally deal with the role of short therapy, there are few randomized controlled trials in the literature that have tried to evaluate its effectiveness, in terms of the recurrence and antibiotic resistance of future infections.

Desai et al. [18] compared the course of short intravenous therapy (up to 7 days) with longer duration cycles in 115 children aged less than two months who were hospitalized for post-UTI sepsis. While not differentiating cystitis from pyelonephritis, the group of patients analyzed included elevated degrees of severity (i.e., complicated infections, typically of the upper urinary tract). The recurrence rate in treated patients lasting less than or equal to 7 days (58 children) was 3%, compared to 7% of patients undergoing prolonged therapy (57 children), for a total of six relapses (two in the short group and four in the standard group). In this study, no information is available regarding oral antibiotic therapy, which can be continued at home; moreover, a duration of 7 days of antibiotic therapy is considered as short therapy, which can actually be assimilated to a standard duration in our study.

A recent review analyzed studies about the difference in UTI recurrence rates after an antibiotic treatment of different duration [19]. Comparing short treatment (2–4 days) and standard treatment (7–14 days), there were no significant differences in the recurrence of infection and, in the case of recurrence, in the appearance of resistant antibiotic bacteria. Another study conducted by Fox et al. [9] retrospectively analyzed 791 patients aged 6 months to 18 years, receiving short- (6–9 days, 297 children) or long-term (greater than 10 days, 494 children) antibiotic therapy for UTIs. The chances of treatment failure (meaning the recurrence of infection, access for symptoms of a UTI at the pediatric emergency room, hospitalization, or death) within 30 days of the discontinuation of antibiotic therapy were similar for patients who were treated with a short course compared to prolonged therapy (11.2% vs. 9.4%; odds ratio 1.22; 95% CI, 0.75–1.98). The authors, having a large number of patients and a recurrence of UTIs, also analyzed the resistance profile of the bacteria responsible for the new infections, showing no significant difference between the two patient groups. Again, unlike our study, there is a different classification of the “short”, including prolonged durations of therapy.

One of the possible explanations for the lower rate of recurrence in children treated with short therapy is the action of systemic antibiotic therapies on the gut microbiota. A study of 35 children under 3 years of age undergoing antibiotics for a UTI [20] showed that the biodiversity of gut bacteria decreases early after only 2 weeks of antibiotic treatment and restores itself about 2 months after the acute event. This condition could lead to the selection of pathogenic bacteria that could migrate from the intestine to the urinary tract and could cause short-term recurrence. In the same study, it was also found that the intestinal biodiversity of patients receiving prophylactic therapy for UTIs with trimethoprim-sulfamethoxazole, for vesicoureteral reflux, does not differ over time from that of children without urinary tract abnormalities; this conclusion is limited to the use of that particular type of antibiotic, and in the literature there are conflicting opinions: as the negative effect on the microbiota of antibiotic prophylaxis is well described [21], starting from the first doses and regardless of dosage, but there is also opposite evidence obtained from pediatric studies on children with urinary tract abnormalities [22].

From our clinical experience, analyzing the 23 children who had a new UTI, we noticed that 73.9% (17) of the relapses were caused by the same bacteria as the first infection; only in 7/17 cases was the antibiogram of the two events the same, while in four cases the appearance of resistance to amoxicillin-clavulanate (in one case associated with a resistance to other antibiotics), in three cases the appearance of resistance to cephalosporins, and three with new resistance to other antibiotics (in one case associated with a resistance to amoxicillin-clavulanate). In eight cases, there was a new resistance to the antibiotic used during the first UTI episode, even if the bacteria were different. Exposure to prolonged doses of drugs could explain reinfections from a bacterium with a worse resistance profile than the first event, but this consideration needs to be explored through future and larger prospective studies.

Importantly, our findings and the other mentioned studies seem to be in line with new evidence about the safety of short therapy in pediatric infectious diseases, such as pneumonia [10]: a recent review considered four clinical trials [23,24,25,26] aimed at analyzing the optimal duration of antibiotic therapy in children affected by non-hospitalized pneumonia, the conclusions of which are the same and indicate that in uncomplicated forms 5 days of antibiotic therapy are sufficient in the eradication of the infection. A similar conclusion can be applied to hospitalized patients [27] from a large multicenter study conducted in 2022, which showed that, in uncomplicated pneumonia cases, a 13–14-day cycle (defined as extended) does not exceed a standard 5–6-day cycle in healing at one month (extended course: n = 127/163, 77.9%; standard course: n = 131/161, 81%). In conclusion, fixed antibiotic durations provide straightforward guidance but do not take into account children’s characteristics or treatment response; a possible approach would be to individualize antibiotic durations via biomarker-assisted guidance such as C-reactive protein or procalcitonin, although these are non-specific indexes for bacterial infections [28].

Our study has some limitations. First, this is a retrospective monocentric study on a relatively small cohort, so the results need to be interpreted cautiously, although they clearly provide a trend worth investigating in larger prospective studies. We also did not perform a subgroup analysis differentiating high from low urinary tract infections, as this is also difficult to establish in young children, although young, hospitalized patients more frequently have a high UTI. The children in our cohort were not randomized and underwent antibiotic therapies of a duration set by physicians. The relatively low number of patients and the single relapse registered in the short therapy group did not allow us to perform a regression analysis. Among the exclusion criteria, we considered patients under one month of age and patients with positive blood culture, thus, not evaluating children who are considered most at risk of developing subsequent complications. We did not run any molecular testing to link the results to trends in antibiotic resistance, and we did not collect data about previous antibiotic exposures earlier in life, which theoretically might have affected the antibiotic sensitivities of the UTI episodes. Finally, due to the small number of relapses in patients with short therapy, we could not compare relapse resistance profiles between the two groups (long versus short), although our results showed that subsequent infections, regardless of the duration of initial therapy, are supported in most cases by germs with a higher antibiotic resistance profile. Last, considering the absence of randomization, Kaplan–Meyer analyses are not the best way to compare those two groups, not considering any other variable. However, it is one of the first studies of its kind involving such a young pediatric population, providing preliminary data that shorter antibiotic regimens may be further studied in larger cohorts of young children admitted with UTIs.

In conclusion, we showed that short antibiotic therapy was equivalent to standard duration therapy for the cure of UTI in hospitalized children and was also associated with a lower rate of recurrences. Given the better understanding of the negative effects of exposure to prolonged antibiotic courses, from alterations of microbiota to the induction of antibiotic resistance, our results reinforce the need to challenge current guidelines on the duration of antibiotic therapy in children hospitalized with UTIs through adequately planned prospective, multicenter, randomized, blinded studies. Importantly, a similar study should be designed for those children with less severe UTIs who do not require hospitalization but are treated in the outpatient setting.

## Figures and Tables

**Table 1:** Clinical information and demographics of the study population.

.	Study Population (N = 112)	Standard Therapy Patients (N = 79)	Short Therapy Patients (N = 33)	*p*
Female (n,%)	46 (41.1%)	31 (39.2%)	15 (45.5%)	0.54
Caucasian ethnicity (n,%)	97 (86.6%)	67 (84.8%)	30 (90.9%)	0.54
Median Age—months (median, IQR)	3 (5)	3 (6)	2 (5)	0.24
Comorbidity (n,%)	15 (13.4%)	8 (10.1%)	7 (21.2%)	0.11
Prematurity (n,%)	1 (0.9%)	0	1 (3%)	0.29
Pathological prenatal renal echography (n,%)	4 (3.6%)	4 (5.1%)	0	0.32
Pathological renal echography during admission for UTI * (n,%)	13 (11.7%)	12 (15.2%)	1 (3.1%)	0.1
Fever (n,%)	110 (98.2%)	77 (97.5%)	33 (100%)	1
Feeding difficulties (n,%)	83 (74.1%)	57 (72.2%)	26 (78.8%)	0.46
Vomiting (n,%)	40 (35.7%)	27 (34.2%)	13 (39.4%)	0.6
Diarrhea (n,%)	21 (18.8%)	14 (17.7%)	7 (21.2%)	0.66
Other Symptoms (n,%)	33 (29.5%)	28 (35.4%)	5 (15.2%)	0.03
Urine test pathological (n,%)	104 (92.9%)	76 (96.2%)	28 (84.8%)	0.05
Pathological WBC count (n,%)	99 (84%)	68 (86.1%)	31 (93.9%)	0.34
Pathological neutrophil count (n,%)	91 (81.3%)	61 (77.2%)	30 (90.9%)	0.11
Pathological lymphocytes count (n,%)	68 (60.7%)	49 (62%)	19 (57.6%)	0.66
Pathological C-reactive protein value ** (n,%)	100 (90.9%)	69 (89.6%)	31 (93.9%)	0.72
Pathological procalcitonin value *** (n,%)	10 (22.7%)	4 (14.8%)	6 (35.3%)	0.11

* performed for 110 patients, ** performed for 110 patients, *** performed for 44 patients.

**Table 2:** Clinical information and demographics of the population divided according to the presence of a relapse of urinary tract infection.

	Non-Relapse Group (N = 89)	Relapse Group (N = 23)	*p*
**Female (n,%)**	39 (43.8%)	7 (30.4%)	0.24
**Age—months (median, IQR)**	3 (6)	3 (6)	0.74
**Caucasian ethnicity (n,%)**	79 (88.8%)	18 (78.3%)	0.19
**Comorbidity (n,%)**	14 (15.7%)	1 (4.3%)	0.3
**Pathological prenatal echography (n,%)**	2 (2.2%)	2 (8.7%)	0.19
**Fever (n,%)**	87 (97.8%)	23 (100%)	1
**Eating difficulties (n,%)**	66 (74.2%)	17 (73.9%)	0.98
**Vomit (n,%)**	32 (36%)	8 (34.8%)	0.92
**Diarrhea (n,%)**	16 (18%)	5 (21.7%)	0.76
**Other symptoms (n,%)**	25 (28.1%)	8 (34.8%)	0.53
***Escherichia coli* UTI (n,%)**	71 (79.8%)	17 (73.9%)	0.57 §
**Pathological WBC count (n,%)**	78 (87.6%)	21 (91.3%)	1
**Pathological neutrophil count (n,%)**	76 (85.4%)	15 (65.2%)	0.04 §
**Pathological lymphocytes count (n,%)**	52 (58.4%)	16 (69.6%)	0.33
Pathological C-reactive protein value * (n,%)	80 (92%)	20 (87%)	0.43
Pathological procalcitonin value ** (n,%)	9 (23.7%)	1 (16.7%)	1

WBC, neutrophil and lymphocytes count were considered normal or pathological according to reference age groups; CRP was considered normal when <5 mg/L; procalcitonin was considered normal when <0.05 ng/mL. § Although the number of observations per cell was never less than 5, we preferred to use the Fisher test in consideration of the presence of expected counts of less than 5. * performed for 110 patients; ** performed for 44 patients.

**Table 3:** Comparison of first urinary tract infection characteristics in the two groups.

	Non-Relapse Group (N = 89)	Relapse Group (N = 23)	*p*
**Germ infection resistant to amoxicillin-clavulanate (n,%)**	15 (16.9%)	4 (17.4%)	1
**Germ infection resistant to cephalosporins (n,%)**	9 (10.1%)	6 (26.1%)	0.08
**Germ infection resistant to empirical therapy performed (n,%)**	9 (10.1%)	4 (17.4%)	0.46

## Data Availability

Not applicable.
